# Editorial: Advances in and applications of predictive toxicology: 2022

**DOI:** 10.3389/fphar.2023.1257423

**Published:** 2023-08-02

**Authors:** Abdulkarim Najjar, Nynke Kramer, Iain Gardner, Thomas Hartung, Thomas Steger-Hartmann

**Affiliations:** ^1^ Beiersdorf AG, Hamburg, Germany; ^2^ Wageningen University and Research, Wageningen, Netherlands; ^3^ Certara UK Limited, Sheffield, United Kingdom; ^4^ Bloomberg School of Public Health, Johns Hopkins University, Baltimore, MD, United States; ^5^ University of Konstanz, Konstanz, Germany; ^6^ Investigational Toxicology, Bayer AG, Pharmaceuticals, Berlin, Germany

**Keywords:** predictive toxicology, new approach methodologies (NAMs), micro-physiological systems (MPS), PBPK modelling, data science

While the first use of the term “Predictive Toxicology” was mainly focusing on *in silico* approaches and applied almost synonymously to computational toxicology (Helma, 2005) it has later been extended to describe models and assays complementary or as replacement to the classical descriptive *in vivo* toxicology (Dearden, 2015). Consequently, FDA’s Predictive Toxicology roadmap published in 2017 lists “new methodologies and technologies to expand FDA’s toxicology predictive capabilities and to potentially reduce the use of animal testing” (FDA, 2017). In December 2022 the FDA Modernization Act 2.0 was passed into US law removing the need for animal testing for every new drug development protocol. Together these regulatory changes offer the possibility of replacing animal testing in drug development with suitably validated Predictive Toxicology methods.

Currently, Predictive Toxicology uses and integrates *in silico*, in chemico and *in vitro* approaches named “New Approach Methodologies (NAMs)” to predict the potential toxic effects of a chemical or drug on living organisms including humans, as well as to assess the safety and potential risks associated with exposure to chemicals, drugs, environmental pollutants, and other substances. Predictive Toxicology forms the backbone of the chemical next-generation risk assessment (NGRA), which integrates NAMs to assure human safety without animal testing (Alexander-White et al., 2022).

The main areas delivering contributions to Predictive Toxicology with probably the greatest recent advancements are micro-physiological systems (MPS) (Roth, 2021), sometimes also termed as advanced cellular models (Pineiro-Llanes et al., 2023), new approaches in data science including analysis of omics data and systems toxicology (Steger-Hartmann et al., 2023), as well as physiologically based pharmacokinetic/toxicokinetic modeling and simulation. The progress in these areas is also illustrated through the manuscripts submitted to this Research Topic of Frontiers in Toxicology:

Cairns et al. present an important milestone in implementing MPS in efficient toxicity testing of drugs. The study describes the development and the implementation of a statistical experimental design approach to a bone marrow MPS, where the results demonstrate an optimal approach to the design of MPS experiments that could be generalizable to other systems and scientific questions. This highlights the impact and the applicability of the MPS in drug safety.

Another implementation of MPS together with physiologically based pharmacokinetic modeling (PBPK) for the chemical safety assessment was reported by Tao et al. The paper describes the development of a skin-liver-thyroid (Chip3) that allows to investigate the interactions between “organs” of interest connected via microfluidic circulation and integrates the typical exposure to chemicals. The MPS model incorporates relevant exposure route (dermal), metabolism in skin and liver, and the biological effects (thyroid hormones) into a single model. The MPS experiments were compiled with PBPK modeling to derive the safe dermal exposure of a chemical in consumer products.

Valls-Margarit et al. describe the comparison of different network-based methods to identify candidate genes involved in adverse events and propose an approach to produce consensus prediction to increase the confidence in the target gene prediction. The findings revealed variations in the performance of the assessed tools against the benchmark and their capacity for providing novel insights into the adverse effects mechanism of the drug.

Gurjanov et al. report, how the reuse of historical data could contribute to the reduction of animal use. Adequately curated and characterized by stringent statistical analyses the historical data could be used to replace or reduce concurrent control groups using so-called virtual control groups, i.e., modeled control groups based on the collected historical data.

With an increasing need to incorporate NAMs in chemical risk assessment and the concomitant need to find alternatives to animal testing, quantitative hazard characterization fundamentally relies on the interpretation of the *in vitro* assay readouts. This requires an extrapolation of the *in vitro* concentration-response, based on an *in vitro* benchmark concentration, into *in vivo* dose−response data; quantitative an *in vitro*-to-*in vivo* extrapolation (QIVIVE). An understanding of the relevant concentration driving the *in vitro* toxicity rather than simply using the applied (nominal) concentration is an important consideration in this step. PBPK modelling integrates the knowledge on the absorption, distribution, metabolism, and excretion (ADME) of a chemical in the human body or organism and provides a means for this extrapolation. In addition, PBPK models facilitate extrapolations across studies, species, routes, and over various exposure scenarios (Najjar et al., 2022). Algharably et al. have reported an implementation of QIVIVE for prediction of *in vivo* prenatal exposure of a chemical leading to developmental neurotoxicity in humans based on *in vitro* toxicity data and discussing several putative neurodevelopmental toxicity mechanisms. The study discusses developing a maternal-fetal PBPK model to perform QIVIVE in a pregnant women population at 15 weeks of gestation.

McNally and Loizou have justified the refinement and calibration of a human PBPK model of a chemical using *in silico*, *in vitro* and human biomonitoring data. The modeling approach demonstrates important implications for the read-across approach, as a part of NAMs for the replacement of animals in chemical safety assessments, to calibrate and validated the developed PBPK model against several data streams from another more data-rich source chemical. The considered read across approach afford more confidence for future evaluations of other similar chemicals.

Together the articles of the Research Topic illustrate the advent of truly disruptive technologies complementing and replacing traditional animal testing in toxicology. Arguably, this is part of an ongoing scientific revolution (Hartung and Tsatsakis, 2021), which promises to make safety assessments faster, cheaper and more human-relevant. The fine contributions within this Research Topic represent steps in this journey.
